# Hemoglobin as an oxygen gasoreceptor

**DOI:** 10.3389/abp.2025.15546

**Published:** 2025-12-17

**Authors:** Savani Anbalagan

**Affiliations:** Institute of Molecular Biology and Biotechnology, Faculty of Biology, Adam Mickiewicz University, Poznań, Poland

**Keywords:** oxygen sensing, oxygen receptor, gasoreceptor, proto-receptor, split-component signal transduction system, gasotransmitter, gasocrine signaling, gasocrinology

## Abstract

In most vertebrates, hemoglobin’s primary function is to transport oxygen and carbon dioxide. Hemoglobin is also expressed in cells such as dopaminergic neurons and chondrocytes, as well as in organelles such as mitochondria. Depending on its location, hemoglobin subunits can interact with proteins involved in various functions, including anion exchange, nitric oxide synthesis, and ATP synthesis. These interactions suggest that hemoglobin has diverse regulatory roles beyond gas transport. During hypoxia and an excess of nitrite and protons, deoxygenated hemoglobin exhibits nitrite reductase activity and produces nitric oxide, a gaseous signaling molecule. Hemoglobin-derived nitric oxide is associated with vasodilation in mammals and the inhibition of mitochondrial respiration in cell cultures. This raises the question of whether hemoglobin functions as a gasoreceptor in these cells or organelles. The HIF1α/PHD2 pathway in mammals and cysteine oxidases in plants are largely responsible for sensing hypoxia, but the identity of oxygen gasoreceptors analogous to the mammalian nitric oxide gasoreceptor soluble guanylate cyclase and the plant ethylene gasoreceptor kinases remains unknown. Since the heme-based dual oxygen-binding and catalytic domain emerged earlier than the allosteric regions, I propose hemoglobin as an oxygen proto-gasoreceptor derivative. Furthermore, since hemoglobin interacts with and regulates proteins depending on its oxygen binding state, I propose that hemoglobin functions as an oxygen gasoreceptor in split-component signal transduction systems. Recognizing hemoglobin as a gasoreceptor will expand the emerging field of gasocrinology to encompass gases that were previously considered primarily metabolic substrates.

## If there is a gasoreceptor for nitric oxide, is there a gasoreceptor for oxygen?

Gases are evolutionarily old signaling molecules and major regulators of cellular metabolism (Aono, 2017; Canfield, 2014). Embryo development is one of the crucial timepoints in which gas homeostasis in cells must be tightly regulated to prevent developmental disorders and embryonic lethality (Maltepe et al., 1997; Froehlich-Santino et al., 2014; Pan et al., 2024). It is conceivable that the diffusion of gases may also provide and regulate positional information and tissue scaling during embryogenesis (Čapek and Müller, 2019; Anbalagan, 2024a). For example, in a few model vertebrate and invertebrate embryos, exposure to an anoxic environment can lead to developmental arrest or suspended animation (Padilla and Roth, 2001; Teodoro and O’Farrell, 2003). Apart from dioxygen (O_2_), an environmentally-derived essential gaseous molecule for vertebrates, other gaseous molecules such as nitric oxide (NO), carbon monoxide (CO), hydrogen sulfide (H_2_S), methane (CH_4_), hydrogen cyanide (HCN), and ethylene (C_2_H_4_) are synthesized in mammalian cells (Carlew et al., 2020; Wang, 2002). The molecular machinery that synthesizes these gases is highly conserved throughout evolution. This suggests the potential importance of these molecules for signaling and maintaining cellular homeostasis throughout an animal’s life. Nevertheless, except for a few gaseous molecules, knowledge about the precise gasoreceptor-mediated signaling role of gases during embryo development is currently lacking both in animals and in plants (Liu et al., 2020; Sonnen and Janda, 2021; Zhang Y. et al., 2023; Corbineau, 2024).

### Gasoreceptors and gasocrine signaling

Since the following sections focus primarily on direct gas-sensing mechanisms, I define gas sensor proteins as proteins that require gases (or their solutes) as substrates for enzyme activity. Gasoreceptors are proteins in which either the binding or lack of binding of a gaseous molecule (or its solute) as a ligand can trigger a cellular signal or response (Anbalagan, 2024a). Gasoreceptors can bind a gaseous molecule either via a metal cofactor or in a metal cofactor-independent manner. Gasoreceptors can be any gas binding-based allosterically-regulated protein with various functions, including enzyme, transcription factor, and ion channel activities (Aono, 2017; Anbalagan, 2024b). For instance, mammalian soluble guanylate cyclase (sGC) and the *Escherichia coli* Direct Oxygen Sensor (DosP) phosphodiesterase are NO and O_2_ gasoreceptors, respectively. While sGC is well known for its role in vasodilation in mammals, DosP regulates *E. coli* biofilm formation and motility (Ignarro and Freeman, 2017; Delgado-Nixon et al., 2000; Shimizu, 2013). Based on the classification of signal transduction systems (STSs) in prokaryotes, the sGC and DosP can potentially be described as a gasoreceptor that function in one-component STSs (Ulrich et al., 2005; Wuichet et al., 2010). However, the classification of sGC is controversial because the cGMP-generating catalytic site forms at the interface of heterodimer. Therefore, a more appropriate classification for sGC could may be as a co-component STS. Finally, diverse cellular signaling events mediated by gases acting as ligands for gasoreceptors are unified under the umbrella term “gasocrine signaling” (Anbalagan, 2024a).

### Unanswered questions in acute O_2_ sensing mechanisms

To the best of my knowledge, the majority of the O_2_-sensing mechanisms in both plants and erythrocyte-containing vertebrates are largely based on O_2_ sensors (Hammarlund et al., 2020). These sensors can belong to major enzyme classes, such as dioxygenases, monooxygenases, and oxidases, all of which require O_2_ as a substrate. However, numerous O_2_-dependent enzymes (more than 200 enzymes in humans alone) can potentially be considered as O_2_ sensors. Depending on their cellular localization, these proteins could potentially perform O_2_-sensing roles in their respective cellular regions or organelles (Li et al., 2023). Amongst these enzymes, Prolyl hydroxylase domain proteins (PHDs) are well-known O_2_ sensor proteins in humans and mice. These enzymes require O_2_ for hydroxylase activity and regulate the Hypoxia-inducible factor 1-alpha/Prolyl Hydroxylase Domain Protein 2/von Hippel-Lindau (HIF1α/PHD2/VHL) pathway (Hammarlund et al., 2020; Li et al., 2023; Ratcliffe and Keeley, 2025). In plants, cysteine oxidases along with the N-degron pathway, and reactive oxygen species-mediated signaling are considered major mechanisms by which plants sense O_2_ (Hammarlund et al., 2020; Holdsworth and Gibbs, 2020).

However, the precise identity of vertebrate and plant O_2_ gasoreceptors similar to the mammalian NO gasoreceptor sGC or the *E. coli* O_2_ gasoreceptor phosphodiesterase (DosP) remains unclear. Even in chemoreceptors cells such as the glomus cells of the carotid body and pulmonary artery smooth muscle cells, the identity of O_2_ gasoreceptors are unknown (Weissmann et al., 2006; Moreno-Domínguez et al., 2020; Gao et al., 2022). In the carotid body, cytochrome c oxidase (complex IV) of the mitochondrial electron transport chain has been reported as an O_2_ sensor (Moreno-Domínguez et al., 2020; Peng et al., 2010; Kumar and Prabhakar, 2012; Peng et al., 2020. Nevertheless, it is unclear whether these sensors bind O_2_ and function as O_2_ gasoreceptors or respond to other accumulating molecules, such as reactive oxygen species, H_2_S, NO, or phosphatidic acid (Gao et al., 2022; Peng et al., 2023; Aragonés et al., 2001). In considering O_2_ *per se* as a gaseous signaling molecule, I proposed it as an essential gasotransmitter, similar to “essential” amino acids (Anbalagan, 2024c).

In the main olfactory epithelium sensory neurons (type B cells) of mice, it has been reported that Soluble guanylate cyclase 1 subunit beta 2 (GUCY1B2) and Transient receptor potential cation channel subfamily C member 2 (TRPC2) mediate calcium influx responses under low O_2_ conditions. However, it is unclear whether these proteins act as O_2_ gasoreceptors (Bleymehl et al., 2016). Similar calcium influx-related responses to acute hypoxia have also been reported in astrocytes, which appear to function independently of peripheral chemoreceptor O_2_-sensing mechanisms. Specifically, in astrocytes isolated from the mice parafacial respiratory group and the retrotrapezoid nucleus, calcium influx is regulated by the differential accumulation of Transient receptor potential cation channel, subfamily A, member 1 (TRPA1) in the plasma membrane under hypoxic conditions (Uchiyama et al., 2020). PHD2 and Neural precursor cell-expressed developmentally downregulated gene 4-1 (NEDD4-1) E3 ubiquitin ligase-dependent trafficking of TRPA1 channels are implicated in this process; however, it is unclear whether these proteins perform the role as O_2_ gasoreceptors. Finally, the “hypoxia sensor” in astrocytes is considered to reside inside the mitochondria, thus suggesting a similar mechanism to that of glomus cells, in which mitochondrial cytochrome c oxidase has been reported as an O_2_ sensor protein, but not an O_2_ gasoreceptor (Moreno-Domínguez et al., 2020; Gao et al., 2022; Angelova et al., 2015). Overall, the identity of O_2_ gasoreceptors in vertebrates and plants remains unknown.

## Oxygen gasoreceptors in one-component signal transduction system

**FIGURE 1:**
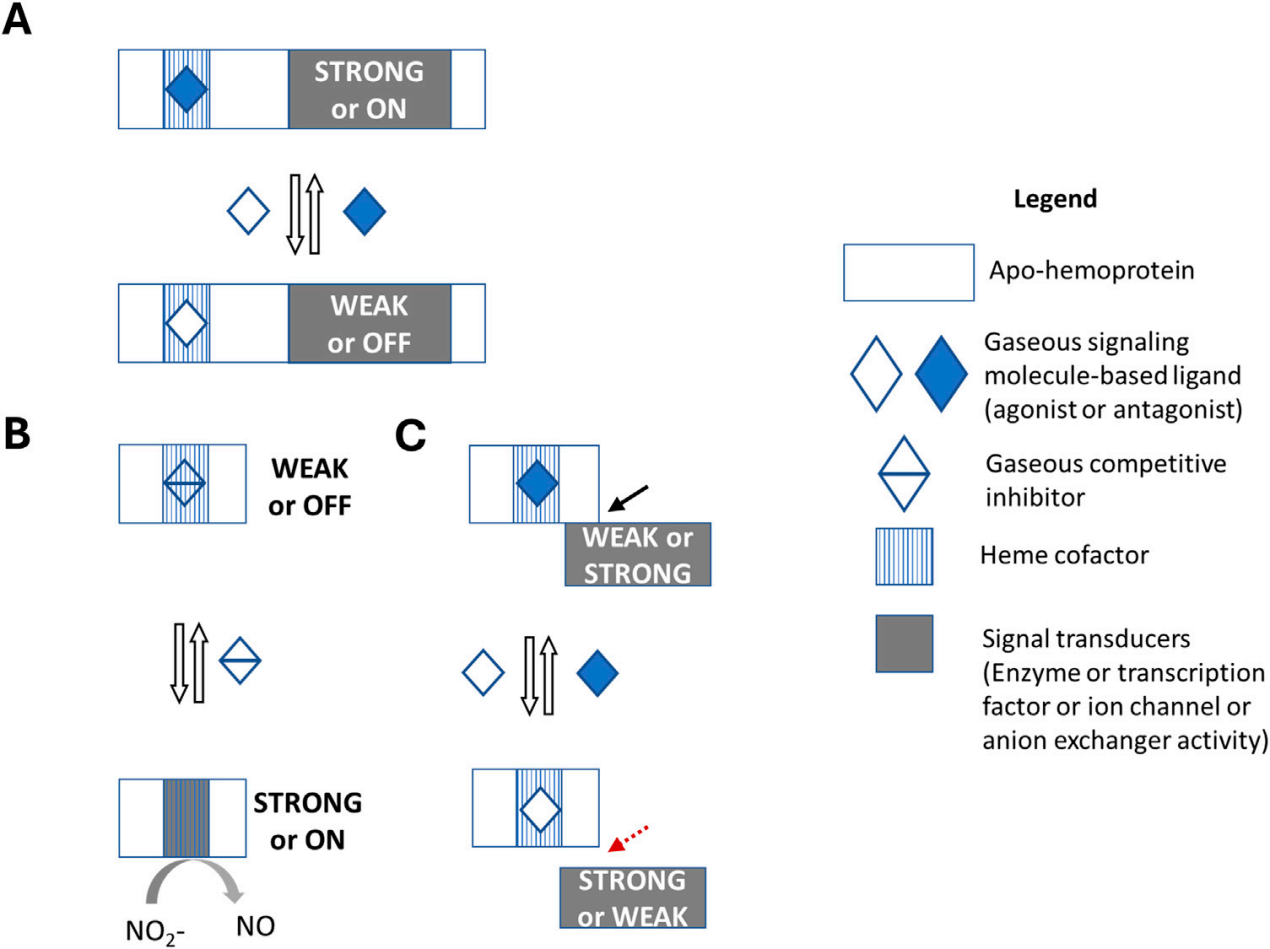
Gasoreceptors in signal transduction systems. Gasoreceptors can bind to gaseous molecules (solutes) in a metal cofactor-dependent or -independent manner. In this scheme, however, gaseous molecule-binding heme cofactor-based gasoreceptors in different signal transduction systems (STSs) are provided. The output domain of the signal transducer in STS can exhibit any type of activity, e.g., an enzyme, a transcription factor, an ion channel, an anion exchanger, etc. **(A)** A gasoreceptor in one-component STS contains two domains: an input domain that binds extracellular or intercellular gaseous molecule, and the second output domain that acts as a signal transducer. Binding of gaseous molecule to the input domain triggers a conformational change, which increases or decreases output domain activity. Examples of O_2_ gasoreceptors in one-component STS include the *E. coli* DosP phosphodiesterase and *C. elegans* GCY-35 soluble guanylate cyclase. **(B)** A proto-gasoreceptor in proto-component STS has one domain where both the input and output domains overlap in a mutually exclusive manner. Thus, the gaseous molecule binding event is competitive inhibition of the intrinsic signal transducer activity of the output domain. Deoxygenated hemoglobin is proposed as a proto-gasoreceptor derivative due to its nitrite reductase activity and the presence of additional allosteric sites. **(C)** A gasoreceptor in split-component STS contains two interacting proteins, one with the input domain and the other with the output domain. The activity of the signal transducer must be dependent on the gas-binding state of the gasoreceptor. Hemoglobin can be considered an O_2_ gasoreceptor and some of its signal transducers include the SLC4A1/BAND3 anion exchanger, deoxygenated hemoglobin with nitrite reductase activity in erythrocytes, eNOS in endothelial cells, and ATP synthase in mitochondria. The black line with an arrow and the red dashed line with an arrow indicate the formation and separation of the complex, respectively. Since additional allosteric regulators can control hemoglobin’s O_2_ binding state, as well as its nitrite reductase activity or interactions with proteins that act as signal transducers, hemoglobin can also be considered a receptor for co-ligands, such as protons and CO_2_ (not shown in the figure).

O_2_ sensors such as *E. coli* DosP phosphodiesterase, *Rhizobium meliloti* FixL kinase, *Caenorhabditis elegans* GCY-35 soluble guanylate cyclase, and *Leishmania major* HemAc-Lm soluble adenylate cyclase, appear to be O_2_ gasoreceptors in one-component STS (Aono, 2017; Delgado-Nixon et al., 2000; Monson et al., 1992; Anbalagan, 2024b) (Figure 1A). These gasoreceptors contain both a heme-based O_2_-binding domain and a signaling domain that exhibits different enzyme activity in each organism, regulating diverse cellular responses. Thus, although “O_2_ sensors” that appear to function as O_2_ gasoreceptors have been reported in various other model organisms, it is surprising that knowledge of vertebrate and plant O_2_ sensing is based on O_2_ sensors and not O_2_ gasoreceptors (Hammarlund et al., 2020; Moreno-Domínguez et al., 2020; López-Barne et al., 2016). Then a few questions arise: Does O_2_ sensing occur only via O_2_ sensors? Are there vertebrate and plant O_2_ gasoreceptors that function as either one-, two-, or multi-component STSs? Or such O_2_ gasoreceptors already been identified but mislabeled, similar to the blind men and the elephant parable (The Blind Men and the Elephant, 1992; Anbalagan, 2023)?

### Androglobin - a candidate O_2_ gasoreceptor

Androglobin (ADGB) which is known to expressed in mammalian testis, female reproductive tract, lungs and brain is a promising candidate for an O_2_ gasoreceptor in one-component STS (Koay et al., 2021; Hoogewijs et al., 2012). The circular permuted globin domain of ADGB can bind O_2_ and NO and based on its binding kinetics, ADGB has been proposed to have an O_2_ sensing rather than transport role (Hoogewijs et al., 2012; Reeder et al., 2024). ADGB is necessary for sperm development in mice and ciliogenesis in cell cultures (Koay et al., 2021; Keppner et al., 2022). Mice ADGB^-/-^ mutants exhibit defective maturation of elongating spermatids, abnormal sperm shape, defects in sperm microtubule and mitochondrial organization (Keppner et al., 2022). However, it is unclear whether ADGB’s protease activity (either autolytic or on its targets) is regulated by its O_2_-binding status. At least for one of its binding partner and protease activity target, Septin 10 (SEPT10), ADGB’s protease activity is unaffected by chronic hypoxia (24 h at 0.2% O_2_) in cell culture-based experiments. Finally, ADGB is not the only O_2_ binding protein that can be considered as a candidate O_2_ gasoreceptor.

## Hemoglobin: a derivative of an ancestral proto-gasoreceptor

### Nitrite reductase activity of deoxygenated hemoglobin

The role of archaeal, bacterial, and yeast protoglobins and flavohemoglobins was proposed to be that of enzymes such as nitrite reductase, NO dioxygenase, denitrosylase, and alkylhydroperoxide reductase, rather than that of gas transport (Zhu and Riggs, 1992; Durner et al., 1999; Bonamore et al., 2003; Corker and Poole, 2003; Freitas et al., 2004; Ascenzi et al., 2014). Similar enzymatic activity has also been reported in plant phytoglobins (Mukhi et al., 2017; Villar et al., 2020). Oxygenated hemoglobin is a NO dioxygenase, while deoxygenated hemoglobin (with 40%–60% oxyHb saturation) and deoxygenated form of its paralogs and orthologs also exhibit nitrite reductase activity. They use co-substrates, such as nitrite and protons, to generate NO, a gaseous signaling molecule and gasotransmitter with a well-known role in vasodilation, among other functions (Huang et al., 2005; Angelo et al., 2008). Given its use of nitrite and protons as a substrate, hemoglobin can be considered one of the nitrite and proton sensor proteins. However, O_2_ and CO are competitive inhibitors of deoxygenated hemoglobin’s nitrite reductase activity and resulting signaling response, thus warranting reconsideration of hemoglobin’s role as a gas-transporting protein.

### Hemoglobin - a gas transporter protein in immobile cells?

Despite its nitrite reductase-dependent signaling role *in vivo*, hemoglobin and its paralogs are only referred to as a transporter or metabolic sensor, not an O_2_ gasoreceptor (Adepu et al., 2024). The well-known role of hemoglobin in transporting O_2_ and its low dissociation constant (K_D_) for O_2_ preclude its classification as an O_2_ gasoreceptor (Hammarlund et al., 2020). However, dissociation constants must be considered not from the perspective of *in vitro* studies but rather from the point of physiological O_2_ concentration *in vivo*, where hemoglobin can be present even in a transient manner. Moreover, reversible binding is not unique to gas-transporting proteins; it is also a feature of previously well-accepted gasoreceptors.

Both hemoglobin and the NO gasoreceptor sGC exhibit reversible binding of O_2_ and NO, respectively (Kharitonov et al., 1997; Winger et al., 2007). sGC binds NO with a relatively higher affinity (dissociation constant K_D_ of 4.2 pM - 54 nM), but dissociates more freely due to its rapid dissociation kinetics. In contrast, O_2_ binds to hemoglobin with a relatively lower affinity (K_D_ of 1–10 μM) and dissociates less freely (Winger et al., 2007; Unzai et al., 1998; Tsai et al., 2012). However, since the concentration of O_2_ in typical vertebrate tissues (5–50 μM) is higher than the concentration of NO (100pM-5 nM), hemoglobin’s relatively low K_D_ for O_2_ should not exclude its consideration as an O_2_ gasoreceptor in a microenvironment-dependent manner (Hall and Garthwaite, 2009). Similar arguments can be made for CO (K_D_ of 1–10 nM) and for other gases that can competitively inhibit hemoglobin (e.g., NO, H_2_S, HCN) (Yuan et al., 2022).

Finally, in vertebrates with erythrocytes, the role of hemoglobin in gas transport largely depends on erythrocytes being passively carried in the bloodstream and O_2_ being delivered to cells due to the Bohr effect (Malte et al., 2021). In human and mice, the NO gasoreceptor sGC is expressed not only in “immobile” vascular smooth muscle cells. Relatively mobile cells, such as platelets, neutrophils, and macrophages, also express sGC (Ciuman et al., 2006; Makhoul et al., 2018). Similarly, hemoglobin is expressed not only in mobile cells, such as mature erythrocytes. Hemoglobin subunit expression has been reported in alveolar epithelial cells, endothelial cells, cortical neurons, A9 dopaminergic neurons in the substantia nigra, cortical and hippocampal astrocytes, oligodendrocytes, epidermal keratinocytes, and chondrocytes and even in organelles such as mitochondria and membraneless condensates (Hedy’s) (Bhaskaran et al., 2005; Biagioli et al., 2009; Richter et al., 2009; Shephard et al., 2014; Brunyanszki et al., 2015; Tahara et al., 2022; Zhang F. et al., 2023; Reed et al., 2025). These findings suggest that, similar to sGC, hemoglobin is expressed in various mobile and immobile cells. However, the precise reason hemoglobin is still accepted as an O_2_ transport protein in relatively immobile cells is unclear. Is it due to hemoglobin’s diffusion or intrinsic mobility within a cell (Kutchai and Staub, 1969; Richardson et al., 2020)? Or is it due to the interaction with other mobile proteins, entropy or the physical activity of immobile cells or animals? Therefore, hemoglobin, a multifunctional protein that transport gases, can also potentially act as a gasoreceptor.

### Hemoglobin as a proto-gasoreceptor derivative

The receptors in one-component STSs are evolutionarily advanced. They have at least two protein domains that perform different functions: one binds the ligand, and the other signals (Ulrich et al., 2005). Since simple gases are evolutionarily ancient than peptides, proto-receptors for gases probably consisted of proteins or nucleic acids with fewer domains (Harold et al., 2023; Anbalagan, 2024b).

The concept of proto-receptors for peptides has been proposed before, but a clear definition has yet to be established (Root-Bernstein, 2005). I define a proto-receptor as an evolutionarily ancient minimalistic receptor type, in which the signaling and sensing domains completely overlap, and which lacks any additional sites for allosteric regulation. Signaling due to proto-receptors can be considered to function in a proto-component STS, that predates one- and split-component STSs. In simple terms, ancestral proteins lacking allosteric regulation can be considered as proto-receptors for the competitive inhibitors that bind and inhibit their activity. For both proto-cells and also modern cells, the absence of a signal can itself serve as a signal. As an analogy, consider a traffic signal that stopped working at a busy intersection. Drivers approaching the intersection would still interpret it as a message to proceed with caution at their own risk. Extending the concept of proto-receptors to gaseous ligands, I define a proto-gasoreceptor as a protein that becomes competitively inhibited by the binding of a gaseous molecule. This results in an absence of cellular signals or responses. O_2_ and hydrogen cyanide (HCN)-binding protein *Campylobacter jejuni* truncated hemoglobin P (Cj-trHbP) has been demonstrated to exhibit peroxidase-like activity *in vitro* (Lu et al., 2007; Bolli et al., 2008; Ascenzi and Pesce, 2017). Thus, Cj-trHbP can be considered as a candidate O_2_ and HCN proto-gasoreceptor in a proto-component STS.

However, the allosteric regulation of protons, CO_2_ and 2,3-Bisphosphoglycerate on mammalian hemoglobin evolved after the heme-containing globin domain had already emerged (Faggiano et al., 2022; Storz, 2025). Therefore, mammalian hemoglobin can be considered as a proto-gasoreceptor derivative (Vinogradov et al., 2006).

### Exogenous nitrite–a bottleneck in hemoglobin’s gasoreceptor classification

In mature erythrocytes in humans, hemoglobin is abundant protein (at 5 mM), making up about 90%–95% of their cellular protein content (Pisciotta and Sullivan, 2008). Along with the erythrocytes, hemoglobin is cyclically carried to hypoxic capillaries, with erythrocyte residence time ranging from sub-second to a few seconds depending on tissue vascularization and blood flow rates (Pittman, 2013). Erythrocyte’s transit time will be further affected by the transit time of other cell types in the capillaries or the pathological state of the vasculature, such as lumen narrowing observed in Atherosclerosis (Wang and Popel, 1993; Hogg et al., 1994). These observation suggest that it could be relatively easier for hemoglobin to be deoxygenated not only in healthy capillaries in physiological conditions but also of vasculature in pathological conditions. In a low-O_2_ environment *in vitro* and under hypoxic conditions *in vivo*, deoxygenated hemoglobin in erythrocytes exhibits nitrite reductase activity, generating NO in a NO synthase-independent manner. This process has also been associated with vasodilation in human subjects, a canine model of hypotonic intravascular hemolysis, and the inhibition of mitochondrial respiration in cell cultures (Brooks, 1937; Nagababu et al., 2003; Basu et al., 2007; Minneci et al., 2008; Gladwin and Kim-Shapiro, 2008; Dent et al., 2021; Helms and Kim-Shapiro, 2013).

Apart from mature erythrocytes, the nitrite reductase activity of the hemoglobin α (HBA) subunit has also been demonstrated in the endothelium of mouse resistance arteries (Keller et al., 2022). Furthermore, endothelial cell-specific conditional *Hba* ^−/−^ mutant mice exhibited reductions in multiple parameters, including nitrite consumption, hypoxia-induced vasodilation, and exercise capacity. These results suggest a physiological role for HBA-derived NO under hypoxic conditions. Similarly, the nitrite reductase activity of myoglobin reduces myocardial infarction in mice via NO upon nitrite treatment; this response is lacking in myoglobin−/− mutants (Hendgen-Cotta et al., 2008).

However, the relatively low rate constant of human hemoglobin (1–10 M^-1^s^-1^), the local concentration of the co-substrate nitrite (200–300 nM in erythrocytes) and endogenous NO scavenging mechanisms, make it difficult to determine whether NO generated from deoxygenated hemoglobin acts as a signaling molecule in physiological or pathological conditions without an exogenous nitrite supply (Dent et al., 2021; Dejam et al., 2005; Wang and Kluger, 2016; Chamchoi et al., 2018).

Overall, at least in laboratory settings, the nitrite reductase activity of human and mouse hemoglobins is insufficient to label hemoglobin as a proto-gasoreceptor-like protein (Figure 1B). Nevertheless, in the case of cyanobacteria *Synechocystis* hemoglobin (SynHb), rice nonsymbiotic hemoglobin (nsHB1), and *Arabidopsis thaliana* phytoglobins (AHb1 and AHb2), which encounter relatively high nitrite concentration *in vivo* and exhibit nitrite reductase activity with relatively high rate constants, such hemoglobin orthologs are more promising candidates for O_2_ proto-gasoreceptor or as O_2_ proto-gasoreceptor derivates (Sturms et al., 2011; Tiso et al., 2012; Kumar et al., 2016). Vertebrates hemoglobin could perhaps act as an endogenous nitrite reductase with receptor-like signaling activity under conditions when nitrite concentration could be higher due to excess of environment-derived nitrite, dietary activities, nitrite enrichment responses, or the lack of homeostasis in the nitrite synthesis pathways (Washio and Takahashi, 2025; Sokal-Dembowska et al., 2025).

## Hemoglobin as an oxygen gasoreceptor in split-component signal transduction systems

In addition to one-component STSs, there are also the examples of two-component STSs, in which the signal transduction occurs via the phosphorylation of a response regulator (Wuichet et al., 2010). These multi-component STSs have also been reported in microbial gas-sensing mechanisms (Lenz and Friedrich, 1998; Buhrke et al., 2004). However, limiting consideration to only one-, two-, or multi-component STS restricts the categories under which gasoreceptors can be classified. As an alternative, I propose the split-component STS.

### Split-component signal transduction system

A split-component STS is a type of cellular signaling mechanism. It involves two proteins: one that constitutes a receptor or input domain, which binds or senses an environmental or intracellular stimulus, and another that constitutes a signal transducer with an output domain, which triggers a response. The ligand-binding status can influence the complex formation and activity of the signal transducer protein. In human and mice, the glucokinase regulator (GCKR, also known as GKRP or glucokinase regulatory protein) and glucokinase are examples of split-component STSs. The glucokinase regulator acts as a receptor for fructose 1-phosphate and fructose 6-phosphate, with glucokinase serving as its signal transducer (Beck and Miller, 2013; Anbalagan, 2025).

The presence of receptor in split-component STS for phosphorylated fructose in mammals raises the question of the identity of O_2_ gasoreceptors acting in split-component STSs (Figure 1C). In theory, any O_2_-binding protein that can physically interact with an enzyme and affect its activity could be considered an O_2_ gasoreceptor in split-component STSs. In this context, the major O_2_-binding proteins hemoglobin, cytoglobin, neuroglobin, and myoglobin could be considered potential O_2_ gasoreceptors and their interacting enzymes could be considered potential signal transducers, provided that the O_2_ binding state-dependent interaction with the enzyme promotes or inhibits the enzyme activity. As detailed in the following sections, hemoglobin interacts with various proteins in the erythrocytes, endothelial cells, and mitochondria. The precise reasons for the hemoglobin subunits’ interaction with proteins that exhibit diverse functions are unclear. Either hemoglobin “hitchhikes” across various proteins while transporting O_2_, or its transport role is regulated. Alternatively, hemoglobin may be an O_2_ gasoreceptor or signal transducer in split-component STS, with some of the interacting proteins acting as signal transducers and receptors, respectively.

### Hemoglobin and SLC4A1 (anion exchanger)

The term “receptor-based signaling” is generally well accepted when a nuclear response is triggered as part of the ligand-receptor activation and signal transduction (Roberts and Kruchten, 2016). However, extracellular and intracellular receptors have been accepted in nuclei-less cells, such as mature erythrocytes and platelets (Tanneur et al., 2006; Vona et al., 2019; Jacobson et al., 2011; Balabin et al., 2018). This suggests that a nuclear response is unnecessary when considering a protein that is strongly allosteric regulated by ligand binding and can trigger a physiological cellular response.

However in mature erythrocytes, if we consider hemoglobin to be an O_2_ gasoreceptor in split-component STS, what is the identity of the signal transducers (Ellsworth et al., 2009)? Another question to consider is what can serve as a signal transducer in split-component STS? Can it only be enzymes, or could it also be other classes of proteins, such as membranal anion exchangers? Since, receptors in one-component STSs can be both ion channels (e.g., mammalian temperature-sensing TRPV ion channels) and enzymes (e.g., *E. coli* O_2_ gasoreceptor DosP phosphodiesterase), why not consider ion channels or anion exchangers as signal transducers (Figure 1C) (Dhaka et al., 2006).

In erythrocytes, hemoglobin binds to solute carrier family 4 member 1 (SLC4A1), also known as band 3 anion exchanger (AE1), in a manner dependent on its O_2_ binding state. This affects the function of SLC4A1 in exchanging bicarbonate (HCO_3_^−^) for chloride (Cl^−^) ions in erythrocytes. This interaction can influence erythrocyte cell physiology including their acid-base balance, metabolic regulation, membrane stability, survival, and lifespan (Chu et al., 2016; Cendali et al., 2024; Jennings, 2021). Therefore, in mature erythrocytes, I propose hemoglobin as an O_2_ gasoreceptor and SLC4A1 as one of its signal transducers in split-component STS.

### Oxygenated hemoglobin and deoxygenated hemoglobin

In mature erythrocytes, another potential signal transducer for hemoglobin in split-component STS is the deoxygenated hemoglobin itself. Heterogenous saturation within hemoglobin tetramers has been reported, and hemoglobin primarily exhibits nitrite reductase activity in its tetrameric form (Huang et al., 2005). The fact that peak nitrite reductase activity occurs at 40%–60% O_2_ saturation of hemoglobin suggests that the oxygenated hemoglobin may act as O_2_ gasoreceptors while the other bound deoxygenated hemoglobin that exhibit nitrite reductase activity could be signal transducer (Huang et al., 2005; Angelo et al., 2008). However, this proposal have the same bottleneck that I proposed earlier in the proto-gasoreceptor derivative section. Another alternative consideration is the role of oxygenated hemoglobin’s NO dioxygenase activity as a signal transducer, and deoxygenated hemoglobin as an O_2_ gasoreceptor in split-component STS. However, the nitrate-specific signaling mechanisms derived from hemoglobin are unclear (Gardner, 2012).

### Hemoglobin and NO synthase

In endothelial cells, a potential signal transducer for hemoglobin in split-component STS is the endothelial NO synthase (eNOS or NOS3), which synthesizes NO. Hemoglobin α and β subunits can interact with endothelial eNOS in mice arterial endothelial cells (Straub et al., 2012; Marozkina et al., 2021). While these interactions seem to play an important role in NO signaling, it is still unknown whether the physical interaction between hemoglobin subunits and eNOS and the activity of eNOS depend on the O_2_ binding state (Straub et al., 2014).

### Hemoglobin β and ATP synthase

In the mitochondria, a potential signal transducer for hemoglobin in split-component STS is the adenosine triphosphate (ATP) synthase (Shephard et al., 2014; Brunyanszki et al., 2015). The physical interaction between hemoglobin β and ATP synthase (ATP5A) has been observed in lysates from rat liver and *Drosophila* mitochondria (Ebanks et al., 2023). However, it is unclear whether the mitochondrial complex formation or dissociation *in vivo* can regulate ATP synthase activity depending on hemoglobin’s binding status to O_2_. Although the role of ATP synthase is well known for its synthesis in ATP, ATP synthase is also a negative regulator of the mitochondrial permeability transition pore, suggesting potential response in the mitochondria (Picard and Shirihai, 2022; Pekson et al., 2023).

Nevertheless, for mitochondrial ATP synthase to be considered a signal transducer, precise knowledge of the intracellular signaling pathways downstream of its products ATP, H_2_O and proton is required (if ATP synthase activity is the only enzyme activity exhibited by ATP synthase). ATP is well known as a substrate for numerous enzymes. Based on the rationale used for O_2_ sensors, many ATP-dependent enzymes can also be considered as ATP sensors (Li et al., 2023). However, most of this signaling will not be considered signaling due to ATP receptors unless the ATP sensor is also strongly allosterically regulated by ATP binding at a site distant from the catalytic site. Currently, only a few extracellular ATP-binding proteins, such as P2X ion channels and P2Y G-protein-coupled receptors, are accepted as ATP or purinergic receptors (Ledderose and Junger, 2020). It is unclear whether similar ATP receptors exist intracellularly in the cytoplasm or various organelles (Fountain et al., 2007). Likewise aquareceptors and proton receptor-mediated signaling pathways too must be considered (Anbalagan, 2024d). Once all these signaling pathways acting downstream of mitochondrial ATP synthase are identified, ATP synthase can be considered a signal transducer in split component-STS.

## Hemoglobin as a receptor for co-ligands - protons and CO_2_

One major one potential pitfall of my proposal regarding hemoglobin as an O_2_ gasoreceptor is the need to distinguish between the roles of O_2_ binding versus hemoglobin’s strong allosteric regulators (protons and CO_2_), which can also favor the deoxyhemoglobin state (Kilmartin and Rossi-Bernardi, 1971; Perrella et al., 1975).

Receptors can exhibit multimodal ligand binding and duality in sensing either for biomolecules or for factors such as photons and temperature (Shen et al., 2011; Assadi-Porter et al., 2018). The majority of the vertebrate hemoglobin with nitrite reductase activity have additional residues that play a significant allosteric regulatory role. Some major allosteric regulators of hemoglobin include protons, CO_2_, bicarbonate, chloride, and organic phosphate anions (e.g., 2,3-bisphosphoglycerate) (Malte et al., 2021; Faggiano et al., 2022; Prange et al., 2001; Ta et al., 2024). As I mentioned earlier, the allosteric regulation by protons and CO_2_ evolved after the emergence of the core heme-binding globin domain responsible for O_2_ binding (Faggiano et al., 2022; Storz, 2025). Protonation and CO_2_ binding in hemoglobin occur at multiple residues, resulting in O_2_ release from hemoglobin (Bohr effect) (Perrella et al., 1975; Gros et al., 1981). Since these allosteric regulatory events must occur collectively to enable hemoglobin nitrite reductase activity, I propose that protons and CO_2_ act as co-ligands for hemoglobin’s gasoreceptor activity. To the best of my knowledge, no studies have tested hemoglobin nitrite reductase activity in wild-type or mutant hemoglobins lacking allosteric regulation by protons, CO_2_-binding, or other biomolecules. Likewise, these allosteric sites must be mutated to test the role of allosteric regulation in interacting with signal transducer-like proteins in split-component STSs. This would allow to determine whether hemoglobin is an O_2_ gasoreceptor or a receptor for multiple ligands, such as protons, CO_2_ and 2,3-bisphosphoglycerate.

## Conclusion

Based on all the arguments presented in this manuscript, I propose hemoglobin as a microenvironment-dependent O_2_ proto-gasoreceptor derivative, as well as an O_2_ gasoreceptor in split-component STS. Similar to hemoglobin, all other O_2_-binding proteins must be considered as putative gasoreceptors, provided that their enzymatic activity, or that of an interacting signal transducer (e.g., enzymes, ion channels, or anion exchangers), is affected by O_2_ binding. A nuclear response is not necessary to consider O_2_-binding proteins as candidate O_2_ gasoreceptors that mediate gasocrine signaling, nor it is necessary for any other class of receptors or signaling in nuclei-less cells. O_2_ concentration and temperature can vary significantly across different tissues, and even within organelles. Therefore, if the dissociation constant (K_D_) values of O_2_ binding are used to exclude certain O_2_-binding proteins from being considered gasoreceptors, these values must be interpreted relative to the physiological O_2_ concentrations and temperatures at which the proteins function *in vivo*. This consideration applies even when O_2_ binding is affected transiently.

The identity of O_2_ gasoreceptors that act upstream of PHDs in hypoxia sensing is unknown. Amongst the numerous signaling molecules, NO is one of the signaling molecule that can act upstream of PHDs during hypoxia. It is tempting to speculate that either hemoglobin or its paralogs may serve as such O_2_ gasoreceptors acting upstream of HIF1α and PHD2 (Metzen et al., 2003; Berchner-Pfannsc et al., 2007). Genetic studies considering the role of hemoglobin or its paralogs as O_2_ gasoreceptors must also consider the possibility that the coding and non-coding nucleic acid sequences of these genes can be potential riboceptors, including gas-sensing riboceptors (Anbalagan, 2024b; Anbalagan, 2024e). Finally, as some of the heme-based gasoreceptors has been proposed to act as aquareceptors, due to the ability of hemoglobin to bind a water molecule at the O_2_-binding site, hemoglobin must also be considered as a proto-aquareceptor derivative or an aquareceptor in split-component STS (Bulone et al., 1993; Colombo et al., 1996; Shadrina et al., 2015; Anbalagan, 2024d).

The discovery of hormones and their functions, the molecular and structural characterization of their receptors and feedback loops, and their medical and diagnostic applications, eventually led to the field of endocrinology (Dittfeld et al., 2017). In the past, NO has been proposed as an endocrine hormone (Bahadoran et al., 2020). Now, a question arises: If the definitive experimental validation of O_2_ gasoreceptors leads to the acceptance of O_2_ as a gaseous signaling molecule and an exogenous, gas-based endocrine hormone? If so, might this lead to a new field of study called gasocrinology? I define gasocrinology as the study of gases, their sources and functions, and disorders affecting cells that exhibit gasocrine and gas-regulated physiological processes. The implications of gasocrinology will likely extend beyond the use of gases in anesthesia in clinics. It will also influence the reconsideration of mechanisms underlying cellular processes, such as metabolic pathways, ferroptosis and the Warburg effect. Additionally, gasocrinology will relate to the theory of consciousness, molecular mechanisms of neurodevelopmental disorders, such as autism spectrum disorder, and neurodegenerative diseases, such as Parkinson’s and Alzheimer’s. Furthermore, gasocrinology will extend to disorders and diseases due to hypoxia and cellular bioenergetics imbalances, and perhaps even to physiological cellular evolutionary mechanisms in a changing environment (Gao et al., 2023; Urbano, 2021; Tokarz and Blasiak, 2014; Wong et al., 2025; Thannickal, 2009).
